# The Effect of Electrospun Scaffold Loaded With Taraxasterol Microspheres on the Proliferation and Differentiation of Osteoblasts

**DOI:** 10.7759/cureus.63989

**Published:** 2024-07-06

**Authors:** Yu Zhang, Ramizu Shaari, Mohamad Arif Awang Nawi, Akram Hassan, Caiyun Cui

**Affiliations:** 1 School of Dental Sciences, Universiti Sains Malaysia, Kelantan, MYS; 2 Department of Oral Medicine, Binzhou Medical University Hospital, Binzhou, CHN

**Keywords:** differentiation, proliferation, osteoblasts, electrospinning, microspheres, taraxasterol

## Abstract

This study aims to observe the effect of electrospun scaffolds loaded with taraxasterol microspheres on the proliferation and differentiation of osteoblasts. Taraxasterol microspheres were prepared by desolventization and electrostatic adsorption technology. The structure of the microspheres was observed under transmission electron microscopy. The drug microspheres were loaded into electrospinning using electrospinning technology, followed by scanning electron microscopy and elemental analysis. Osteoblasts were cultured in vitro, and the effects of drug carriers on osteoblast proliferation and differentiation were observed through Cell Counting kit 8 (CCK-8) cell proliferation detection and alkaline phosphatase activity detection. Transmission electron microscopy showed that the prepared drug microspheres have a double-layer structure, which can effectively reduce the sudden release of drugs. The electrospinning had a porous three-dimensional structure between them, which was conducive to cell adhesion. After loading microspheres, there was a significant difference in electrospinning diameter and nodular protrusions, energy dispersive spectroscopy (EDS) elemental analysis showed that the proportion of nitrogen elements increased significantly after the addition of microspheres, a CCK-8 detection showed that drug carrier scaffolds loaded with taraxasterol had a promoting effect on osteoblast proliferation (P<0.05). Alkaline phosphatase detection showed that drug carrier scaffolds loaded with taraxasterol can promote early differentiation of osteoblasts (P>0.05). Electrospinning loaded with taraxasterol microspheres can promote osteoblast adhesion, proliferation, and differentiation.

## Introduction

Taraxasterol is a traditional Chinese medicine extract [1]. However, its poor water solubility limits its anti-inflammatory [2] and antitumor effects [3]. In the early stage, our experimental team also confirmed that taraxasterol can protect osteoblasts in high-glucose environments, and drug carriers can load drugs while increasing drug utilization [4]. Simple drug carrier adsorption may cause sudden drug release, which cannot effectively achieve drug-sustained release. Therefore, how to make drugs sustained-release for a long time is a research hotspot for many scholars [5]. Drug-loaded microspheres are nanoscale drug carriers, especially bilayer-structured microspheres, which can effectively encapsulate drugs and prevent sudden drug release [6,7]. Electrospinning technology has also been a hot topic in recent years for the preparation of drug carriers, with many applications in fields such as biological tissue engineering, environmental protection, and drug development [8]. The carriers prepared by electrospinning are mostly in the form of membranes, which are easy to cut and modify, and can achieve fixed-point attachment drug delivery, enabling local drug application [9].

This project will use desolventization and electrostatic adsorption technology to create taraxasterol microspheres using biocompatible and widely available raw materials: fetal bovine serum protein and chitosan. The electrospinning technique was used to mix drug-loaded microspheres with polycaprolactone (PCL) to create electrospun thin films. The study found that drug carriers affect the proliferation and differentiation of MC3T3-E1 cells, providing evidence for clinical bone protection and augmentation.

## Materials and methods

Preparation of drug microspheres

We added the appropriate amount of taraxasterol to a bovine serum albumin (BSA) solution. Then, at a rate of 30 mL/hour, we added 40 mL of anhydrous ethanol to the BSA aqueous solution. The chitosan solution was prepared by adding the appropriate amount of chitosan to an acetic acid solution. To make a stable chitosan-coated nanosphere structure, we slowly injected 40 mL of chitosan solution into the BSA solution, mixed it for eight hours, freeze-dried the powder, collected the supernatant after each centrifugation, and stored it at 4°C.

Transmission electron microscopy observation

Before freeze-drying, we immersed the nanoparticle suspension in 1 mL of 50% ethanol. When the suspension was well blended, we took a small amount and expanded it 200 times with anhydrous ethanol. Took one drop of the enlarged liquid and placed it on a copper mesh coated with carbon. Placed it in a sterile ventilation vent for air drying and tested using TEM. Each sample should be evaluated in three parts.

Preparation of electrospinning

Microsphere nanoparticles (NP)/PCL group: we weighed polycaprolactone and dissolved it in dichloromethane (DCM) in a glass bottle. We then dissolved the freeze-dried microsphere powder in N, N-dimethylformamide (DMF); stored it overnight with a magnetic heating stirrer to obtain an electrospinning solution; and used a microinjection pump to fix a 10 mL syringe, which had a drawn electrospinning solution. We connected the needle to a PVC tube, connected the tip of the needle to the DC high-voltage positive electrode, connected the receiving plate to the high-voltage negative electrode, and ground the wire. The operating parameters were high voltage (25.0 ± 0.5) kV, flow rate of 3 mL/hour, and receiving distance of 20 cm. We spun electrospinning under suitable humidity and room temperature conditions. After electrospinning, we placed the tin foil in a vacuum-drying oven to dry for 24 hours and prepared electrospun scaffolds of the PCL group using the same method.

Scanning electron microscopy observation and energy dispersive spectroscopy (EDS) analysis

We cut the film into 5 mm x 5 mm sizes and sequentially bonded it onto the metal loading platform. Then, we sprayed gold collected images under 20 kV and 500 times magnification and performed EDS analysis.

Osteoblast passage and culture

We used MC3T3-E1 cells. Configuration containing 10% FBS and 1% penicillin/streptomycin α-MEM medium was placed in a cell culture incubator with 5% CO2 and 37°C for cultivation. After two days of medium exchange, the experiment was conducted for three passages during the logarithmic growth phase of the cells.

CCK-8 cell proliferation detection

We inoculated MC3T3-E1 cells at a density of 3 × 104 cells/mL onto a 96-well plate and divided them into two groups: PCL and NP/PCL. Subsequently, we placed the corresponding carrier scaffolds in the wells, with each well containing 200 μL cell suspension cultured for one day, four days, and seven days. The original culture medium was discarded, and a CCK-8 mixture was added according to the instructions. The absorbance (OD) value of each well was detected by setting the wavelength of the enzyme-linked immunosorbent assay (ELISA) reader to 450 nm.

Alkaline phosphatase activity detection

We inoculated cells in a 6-well plate with a cell suspension of 3 × 104 cells/mL and grouped them as before. We set up three wells of 1 mL cell suspension per well and incubated the cells in a constant temperature incubator for 24 hours. We changed the culture medium containing calcium induction medium and incubated it for one day, four days, and seven days, respectively. We covered the cells with cell lysis solution in each well, fully lysed them, centrifuged them, and took the supernatant. We referred to the instructions of the reagent kit and used an ELISA to detect the OD value at a wavelength of 405 nm.

Statistical analysis

The Statistical Product and Service Solutions (SPSS, version 21.0; IBM SPSS Statistics for Windows, Armonk, NY) statistical software was used for analysis and processing, represented by means ± standard deviation (SD). The comparison of intergroup differences was conducted using a one-way analysis of variance, and P<0.05 was considered statistically significant.

## Results

**Figure 1:**
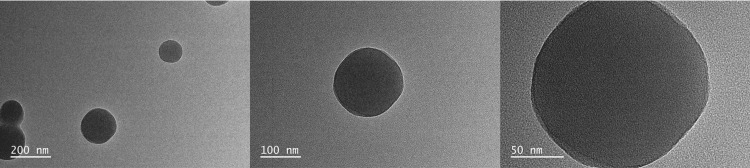
Taraxasterol microspheres have a smooth double-layer spherical structure

Observation of taraxasterol microspheres with double-layer spherical structure by transmission electron microscopy (Figure 1).

Transmission electron microscopy observation showed that the prepared microspheres had a relatively uniform particle size and a double-layer spherical structure with an outer layer wrapped in chitosan.

Scanning electron microscopy (SEM) observation showed that the electrospinning carrier had a three-dimensional porous structure, and there was a significant difference in N elements after the addition of microspheres.

**Figure 2:**
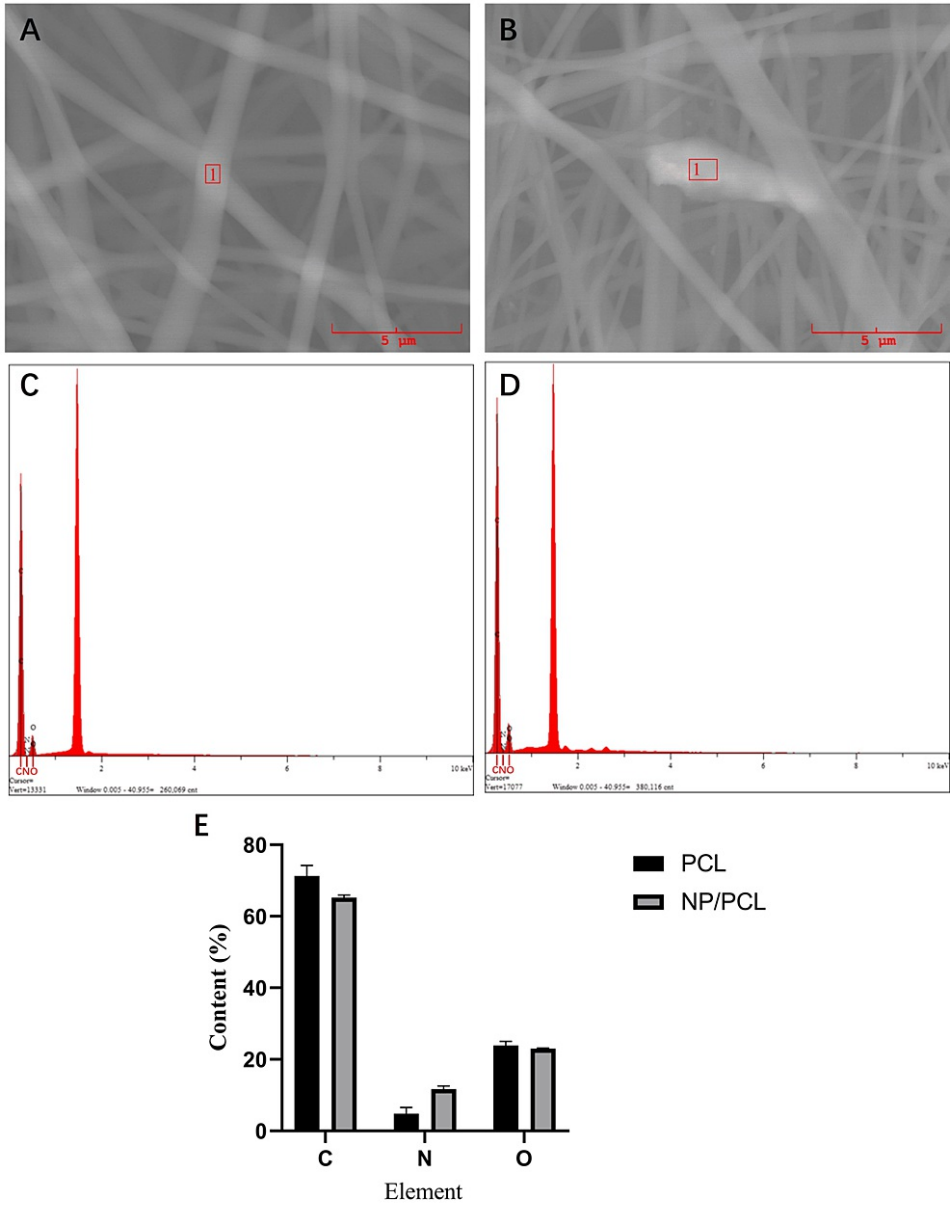
Scanning electron microscopy shows that the electrospinning carrier is a loose porous scaffold (A) SEM showed that the spinning diameter of the PCL group is relatively uniform and smooth. (B) SEM showed a significant difference in spinning diameter and the presence of nodules in the NP/PCL group. (C) Composition of elements C, N, and O in the PCL group. (D) Composition of elements C, N, and O in the NP/PCL group. (E) Differences in elements between PCL and NP/PCL groups.

SEM observation showed that the fiber diameter of the PCL group scaffold was more uniform, and the surface was smoother (Figure 2). There was a significant difference in the fiber diameter of the NP/PCL group scaffold, with some fibers showing particle protrusions. Both had a loose and porous 3D structure, which was conducive to cell attachment and provided growth space for cell growth. EDS elemental analysis showed that the proportion of N element increased after the addition of microspheres.

The addition of taraxasterol microspheres can promote the proliferation of MC3T3-E1 cells.

**Figure 3:**
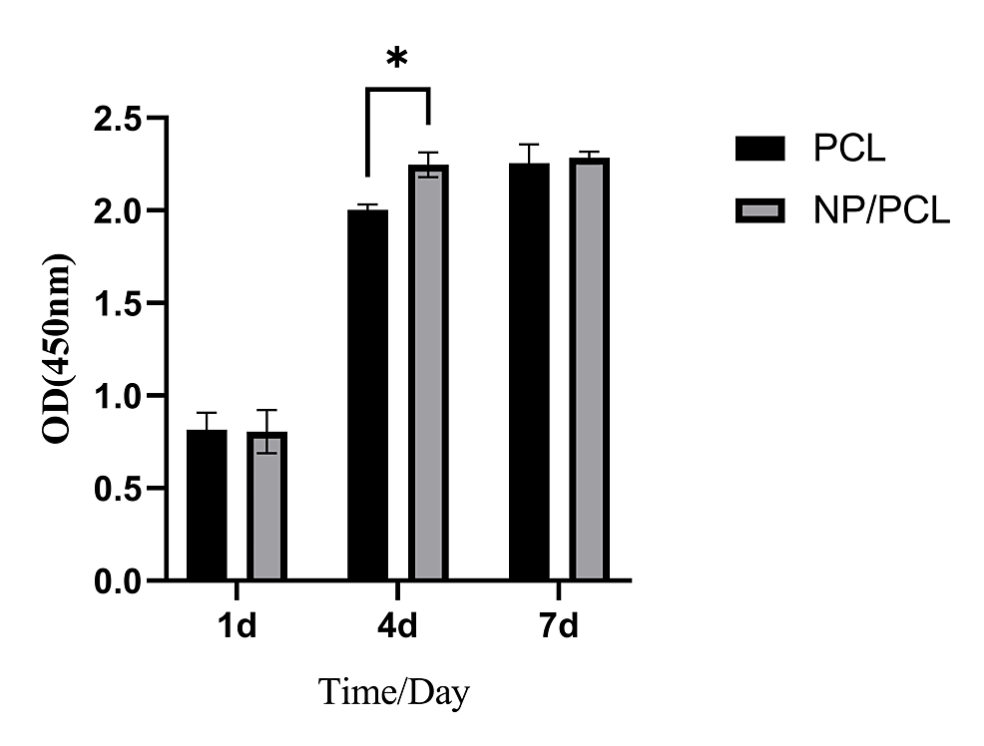
Results of osteoblast proliferation detection * At four days, the NP/PCL group showed better cell proliferation activity compared to the PCL group, and the difference was statistically significant (P<0.05).

After one day of cultivation, there was no statistically significant difference in bone cell proliferation between the PCL and NP/PCL groups (P > 0.05). After four days of incubation, the NP/PCL group showed considerably higher cell proliferation than the PCL group (P<0.05). Cultivate for seven days. There was no statistically significant difference in bone cell proliferation between the PCL and NP/PCL groups (P>0.05). This is presented in Figure 3.

Taraxasterol microspheres added can promote early differentiation of MC3T3-E1 cells.

**Figure 4:**
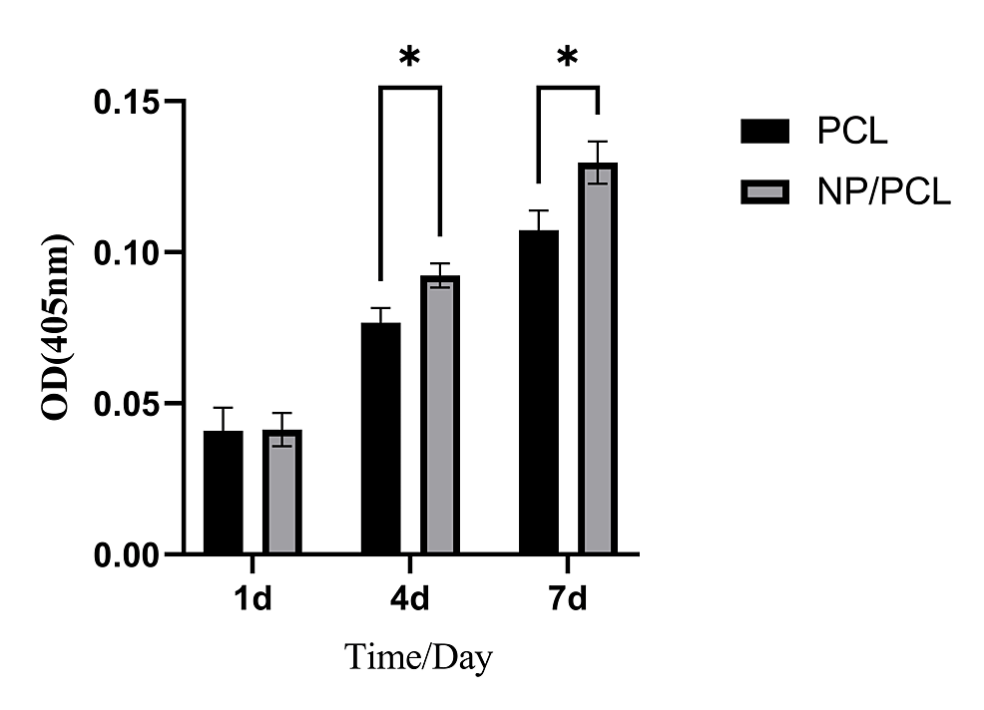
Alkaline phosphatase activity test results *At 4d and 7d, the NP/PCL group showed better alkaline phosphatase activity compared to the PCL group, and the difference was statistically significant (P<0.05).

After one day of cultivation, there was no significant difference in alkaline phosphatase activity between the PCL and NP/PCL groups (P>0.05). However, after four and seven days of cultivation, the NP/PCL group demonstrated a significant increase in alkaline phosphatase activity compared to the PCL group (P<0.05). This is presented in Figure 4.

## Discussion

Taraxasterol has anti-inflammatory, antitumor, and antioxidant stress effects [10], our experimental team has also confirmed in the early stage that taraxasterol has a protective effect on osteoblasts in high-glucose environments. However, natural plant extracts have the disadvantage of poor water solubility, which limits their biological activity [11]. Drug carriers have been a hot research topic in recent years. There are various types of drug carriers, such as biological agents, chemically synthesized scaffolds, and so on [12]. A good drug carrier should have good biocompatibility, drug adsorption, biodegradability, promote the growth of osteoblasts, and provide space for the growth of osteoblasts [13]. PCL is a hydrophobic aliphatic polyester based on hydroxyalkanoic acid, which has advantages such as good biocompatibility, biodegradability, and no rejection to the body [14]. It is currently a commonly used synthetic polymer material and has been approved by the US Food and Drug Administration for clinical use. It is often used as a drug carrier and in bone tissue engineering [15]. The PCL film prepared by electrospinning technology is a scaffold material recently studied by members of our experimental team. It has been confirmed that its loose and porous three-dimensional space is conducive to cell growth [16]. Nanomicrospheres are currently widely used in drug delivery and other fields, as they can effectively encapsulate drugs and achieve sustained drug release [17]. This experiment innovatively combines drug microspheres with electrospinning technology to construct a dual sustained-release drug carrier, providing conditions for the clinical application of taraxasterol.

In this experiment, taraxasterol microspheres were prepared by desolventization and electrostatic adsorption technology. Transmission electron microscopy showed that the prepared microspheres were double-layer spherical structures with relatively uniform particle size, and the outer layer was wrapped with chitosan, which can effectively alleviate the sudden release of drugs. The taraxasterol microspheres were combined with PCL by electrospinning technology to prepare electrospinning films. SEM showed that the prepared electrospinning film was a loose and porous three-dimensional structure, which could provide growth space for cell attachment and proliferation [18]. After the inclusion of microspheres, the electrospinning diameter became uneven, the surface roughened, and nodular protrusions developed. This could be caused by microspheres becoming stuck in the electrospinning machine's needle or uneven liquid tassels [19]. In the cell proliferation test, at four days, the addition of microspheres can significantly promote the proliferation of MC3T3-E1, and the promotion effect is not obvious at one day. The reason may be that the multi-void carrier structure of the PCL group also provides space for cell attachment, and the cells attach and grow well. The reason for the no difference at seven days may be that the cell proliferation is more contact-inhibited, so the difference is not large. Alkaline phosphatase detection found that the alkaline phosphatase activity of the NP/PCL group increased significantly at four and seven days, suggesting that drug microspheres can promote the early differentiation of osteoblasts.

This technique combines medication microspheres and electrospinning technologies in a unique way to undertake early carrier investigation. Taraxasterol has been proven to increase osteoblast proliferation and differentiation. However, this experiment is simply a preliminary investigation of the performance of synthetic drug carriers. The basic performance of drug transporters is assessed, and the detection signs are insufficiently broad. Element detection can only reflect the initial loading of microspheres into electrospinning and not the loading amount or drug release ability. Afterward, this experimental team will conduct more detailed tests, such as microsphere particle size analysis, electrospinning film surface properties, drug-sustained release, and so on, to provide more detailed data support for the clinical application of taraxasterol microsphere electrospinning film.

## Conclusions

In this experiment, double-layer microspheres were successfully prepared by deionization and electrostatic adsorption technology and spun into electrospinning to form a loose and porous three-dimensional structure, which is conducive to the proliferation and attachment of cells. The electrospinning carrier of taraxasterol microspheres was applied to osteoblasts, and it was found that the microsphere carrier can significantly promote the proliferation and differentiation of osteoblasts. The preparation of taraxasterol drug carriers has achieved initial success, and our team will conduct a more comprehensive exploration of more performance.
